# Pragmatic criteria of the definition of neonatal near miss: a comparative study

**DOI:** 10.11606/S1518-8787.2017051006587

**Published:** 2017-11-22

**Authors:** Pauline Lorena Kale, Maria Helena Prado de Mello Jorge, Ruy Laurenti, Sandra Costa Fonseca, Kátia Silveira da Silva

**Affiliations:** I Universidade Federal do Rio de Janeiro. Instituto de Estudos em Saúde Coletiva. Área de Epidemiologia e Bioestatística. Rio de Janeiro, RJ, Brasil; II Universidade de São Paulo. Faculdade de Saúde Pública. Departamento de Epidemiologia. São Paulo, SP, Brasil; III Universidade Federal Fluminense. Instituto de Saúde Coletiva. Departamento de Epidemiologia e Bioestatística. Niterói, RJ, Brasil; IV Fundação Oswaldo Cruz. Instituto Nacional de Saúde da Mulher, da Criança e do Adolescente Fernandes Figueira. Departamento de Epidemiologia Clínica. Rio de Janeiro, RJ, Brasil

**Keywords:** Near miss, classification, Infant Mortality, Birth Wieght, Infant, Premature Apgar score, *Near miss*, classificação, Near miss, Mortalidade Infantil, Peso ao Nascer, Recém-nascido, Prematuro e Índice de Apgar

## Abstract

**OBJECTIVE:**

The objective of this study was to test the validity of the pragmatic criteria of the definitions of neonatal near miss, extending them throughout the infant period, and to estimate the indicators of perinatal care in public maternity hospitals.

**METHODS:**

A cohort of live births from six maternity hospitals in the municipalities of São Paulo, Niterói, and Rio de Janeiro, Brazil, was carried out in 2011. We carried out interviews and checked prenatal cards and medical records. We compared the pragmatic criteria (birth weight, gestational age, and 5’ Apgar score) of the definitions of near miss of Pileggi et al., Pileggi-Castro et al., Souza et al., and Silva et al. We calculated sensitivity, specificity (gold standard: infant mortality), percentage of deaths among newborns with life-threatening conditions, and rates of near miss, mortality, and severe outcomes per 1,000 live births.

**RESULTS:**

A total 7,315 newborns were analyzed (completeness of information > 99%). The sensitivity of the definition of Pileggi-Castro et al. was higher, resulting in a higher number of cases of near miss, Souza et al. presented lower value, and Pileggi et al. and de Silva et al. presented intermediate values. There is an increase in sensitivity when the period goes from 0–6 to 0–27 days, and there is a decrease when it goes to 0–364 days. Specificities were high (≥ 97%) and above sensitivities (54% to 77%). One maternity hospital in São Paulo and one in Niterói presented, respectively, the lowest and highest rates of infant mortality, near miss, and frequency of births with life-threatening conditions, regardless of the definition.

**CONCLUSIONS:**

The definitions of near miss based exclusively on pragmatic criteria are valid and can be used for monitoring purposes. Based on the perinatal literature, the cutoff points adopted by Silva et al. were more appropriate. Periodic studies could apply a more complete definition, incorporating clinical, laboratory, and management criteria, including congenital anomalies predictive of infant mortality.

## RESUMO

**OBJETIVO:**

Testar a validade dos critérios pragmáticos de definições de *near miss* neonatal, estendendo-as para todo o período infantil, e estimar indicadores de assistência perinatal em maternidades públicas.

**MÉTODOS:**

Constituiu-se uma coorte de nascidos vivos de seis maternidades dos municípios de São Paulo, Niterói e Rio de Janeiro em 2011. Foram realizadas entrevistas e consultas aos cartões de pré-natal e prontuários. Critérios pragmáticos (peso ao nascer, idade gestacional e escore de Apgar 5’) das definições de *near miss* de Pileggi et al., Pileggi-Castro et al., Souza et al. e Silva et al. foram comparados. Foram calculados sensibilidade, especificidade (padrão-ouro: óbito infantil), percentual de óbitos entre recém-nascidos com ameaça à vida e taxas de *near miss*, mortalidade e desfechos graves por 1.000 nascidos vivos.

**RESULTADOS:**

Foram analisados 7.315 recém-nascidos (completude das informações > 99%). A sensibilidade da definição de Pileggi-Castro et al. foi maior, resultando em um maior número de casos de *near miss*; a de Souza et al. apresentou menor valor, e as de Pileggi et al. e de Silva et al. apresentaram valores intermediários. Há um aumento da sensibilidade quando se estende o período de 0–6 para 0–27 dias, e um declínio quando se amplia para 0–364 dias. Especificidades foram elevadas (≥ 97%) e superiores às sensibilidades (54% a 77%). Uma maternidade de São Paulo e outra de Niterói apresentaram, respectivamente, as menores e as maiores: taxas de mortalidade infantil, *near miss,* e frequência de nascimentos em situação de ameaça à vida, independentemente da definição.

**CONCLUSÕES:**

As definições de *near miss* baseadas exclusivamente em critérios pragmáticos são válidas e podem ser utilizadas para fins de monitoramento. Com base na literatura perinatal, os pontos de corte adotados por Silva et al. foram mais adequados. Estudos periódicos poderiam aplicar uma definição mais completa, com incorporação de critérios clínicos, laboratoriais e de manejo, incluindo as anomalias congênitas preditoras do óbito infantil.

## INTRODUCTION

Neonatal near miss happens when a newborn who presented a severe complication in the first days of life almost died but survived during the neonatal period^13^. There is still no standard definition of the criteria to classify this event. In analogy to the definition of maternal near miss^19^, “severe complications” are identified – conditions that threaten the life of the newborn –, and the survival from these conditions defines neonatal near miss^13^ ^,^ ^14^ ^,^ ^16^.

Despite the decline in infant mortality in Brazil, 26,723 deaths were recorded in the neonatal period in 2014^a^. Considering that approximately four cases of near miss occur for one neonatal death, the application of the definition of near miss will allow the identification of a greater amount of risky births^2^, which represents an advantage for studies of associated factors, as it increases their power^2^ ^,^ ^16^, particularly when neonatal mortality is reduced^2^.

Based on the concept of neonatal near miss, indicators that express the burden of newborns at risk (severe morbidity and mortality) can also be estimated, subsidizing both the calculation of the resources needed for health services and the evaluation of the quality of care provided^13^ ^,^ ^20^.

The World Health Organization (WHO) has proposed a protocol with several criteria as markers of neonatal near miss, classified into categories of indicators of organic, clinical, or laboratory dysfunction, indicators of management of severity, and conditions of the newborns – weight, gestational age, and asphyxia – associated with greater severity in the neonatal period^20^.

Low birth weight, prematurity, and asphyxiation are conditions strongly associated with neonatal deaths^2^ ^,^ ^9^, and different cutoff points of birth weight, gestational age, and fifth-minute Apgar scores have been studied in the composition of pragmatic criteria for the definition of “threat to life” and, later, neonatal near miss^13^ ^,^ ^14^ ^,^ ^16^ ^,^ ^20^. They are considered pragmatic by the wide availability of information in medical documents and databases of the health sector^13^, besides being validated, associated or not with other criteria, to identify neonatal near miss^13^ ^,^ ^14^ ^,^ ^16^.

Pileggi et al.^13^ have validated a pragmatic and operational definition of neonatal near miss in Brazilian maternity hospitals^b^, considering only the information on gestational age, weight at birth, and fifth-minute Apgar score. Data from two large WHO surveys were used to validate the definition of neonatal near miss by comparing the pragmatic criteria of the above definitions and including, in a new definition, in addition to conditions of the newborn, management markers^14^. The study “Nascer no Brasil”, of hospital basis and national representativity, has validated the definition of neonatal near miss, including markers of the conditions of the newborn, use of mechanical ventilation, and presence of congenital anomaly^16^. The differences between the criteria based on birth weight, gestational age, and fifth-minute Apgar score of the four definitions of near miss of the mentioned studies are the cutoff points. Only the study of Silva et al.^16^ has analyzed the entire neonatal period (0–27 days) and not only the early neonatal period (0–6 days).

The identification of the set of pragmatic criteria that better captures newborns with severe neonatal morbidity (conditions that threaten life) enables the application of these criteria in different socioeconomic contexts and in the absence of technologies applied to care.

In order for the concept of near miss to be an effective instrument for improving maternal and child health, we need to identify cases of near miss and analyze the corresponding indicators^20^.

In this article, we tested the validity of the pragmatic criteria of the existing definitions of neonatal near miss, using them throughout the infant period, and we estimated indicators of perinatal care, related to the concept of neonatal near miss, in public maternity hospitals.

## METHODS

We performed a cohort of live births (LB) from a cross-sectional study in maternity hospitals of the Brazilian Unified Health System (SUS) with high frequency of live births in the municipalities of São Paulo, State of São Paulo, and Niterói and Rio de Janeiro, State of Rio de Janeiro, in three months of the second half of 2011. The four of the largest maternity hospitals in São Paulo are: one philanthropic (A), the only one that is not a reference for high-risk pregnancies; one national reference in Women’s and Newborn’s Health (B); one belonging to a university hospital (C); and another reference for high-risk pregnancy and parturient and neonatal in the Metropolitan region of Greater São Paulo (D). The selected maternity hospital of the municipality of Rio de Janeiro (E), located in the Metropolitan Region I, covers the local population, and the maternity hospital of Niterói (F) is a reference for low- and high-risk pregnancy of the Metropolitan Region II of the state of Rio de Janeiro.

This study is a result of the integration of research studies on the health of women hospitalized for abortion or delivery and their newborns in the municipalities of São Paulo (FAPESP – Notice PPSUS 2009), Rio de Janeiro, and Niterói (Ministry of Health/Work Education for Health Program – PET Health/Health Surveillance – Notice 7 2010 and CNPq – Notice 20/2010). We used common instruments for data collection and study logistics.

Primary data was collected in an interview with the parturient 12 hours after delivery by previously trained health students. In addition, we consulted hospital records, the pregnancy card, and the delivery room record. Particularly the information on birth weight and Apgar score were obtained from hospital records. In the maternity hospitals of São Paulo, information on gestational age was obtained mainly from hospital records without reference to the method of calculation or estimation, following the protocols of the physicians who cared for the parturient. In Rio de Janeiro, the method used to calculate or estimate gestational age followed an algorithm that prioritized different data sources, in this order: the date of the last period (DLP), when compatible with ultrasound (US) before 20 weeks; US, when the values of DLP were inconsistent or ignored; finally, neonatal clinical examination. The information was worked as complete weeks of gestation.

We selected 7,362 LB for analysis, and approximately 1% of the records were excluded because they did not present any of the data on birth weight, gestational age, or fifth-minute Apgar score. Therefore, the study population consisted of 7,315 LB, of which 5,535, 1,222, and 557 LB were from the maternity hospitals of São Paulo, Rio de Janeiro, and Niterói, respectively.

Data on infant mortality after hospital discharge were obtained from the Mortality Information Improvement Program (PRO-AIM) of the Municipality of São Paulo and from the Mortality Information System (SIM) of the State Department of Health of Rio de January (probabilistic relationship of databases).

The pragmatic criteria – markers of life threatening conditions of the newborn – and the cutoff points that support each of the four selected definitions of near miss are based on birth weight, gestational age, and fifth-minute Apgar scores (Table 1) .

**Table 1:** Situations that threaten the lives of newborns: pragmatic criteria of definitions of neonatal near miss*.

Definitions of near miss*	Pragmatic criteria		

	Birth weight (grams)	Gestational age (weeks)	Fifth-minute Apgar score
Pileggi et al.^13^	< 1.500	< 30	< 7
Pileggi-Castro et al.^14^	< 1.750	< 33	< 7
Souza et al.^20^	< 1.500	< 31	< 5
Silva et al.^16^	< 1.500	< 32	< 7

* The definitions of near miss will be named in this comparative study by the surname of the first author.

The validation of the definition of neonatal near miss with Brazilian data has been performed by Pileggi et al.^13^ (0–6 days) and Silva et al.^16^ (0–27 days). The validation process, regardless of criteria, uses neonatal deaths as the gold standard.

The only definition of near miss based exclusively on pragmatic criteria is the one by Pileggi et al.^13^ The other ones include clinical, laboratory, and management criteria, and the one by Silva et al.^16^ also includes the presence of congenital anomalies. When different cutoff points are compared, the choice lies on the combination of higher sensitivity and lower heterogeneity^14^. Only Souza et al.^20^ have presented the definition of near miss without a validation study.

In this study, we analyzed only the pragmatic criteria of each definition of near miss by the infant age component (early neonatal: 0–6, neonatal: 0–27, and infant: 0–364 complete days).

We calculated the frequencies of the four life-threatening definitions evaluated (with the presence of at least one of the pragmatic criteria). The study population was described in relation to situations that threaten the lives of newborns, severe morbidity (near miss), and infant mortality. In addition, we estimated the frequencies of missing or ignored information of the variables: birth weight, gestational age, and fifth-minute Apgar score.

For the validation of the definitions of near miss based only on pragmatic criteria, we calculated sensitivity, specificity (considering infant mortality by the age component as the gold standard), and respective confidence intervals (95%CI). Regarding the choice of the best set of criteria, we evaluated the combination of sensitivity and specificity, the performance in the evaluation of the quality of the care, and, based on the literature, the adequacy of cutoff points used in perinatology. The definitions of near miss were named in this comparative study by the surname of the first author.

Considering the proposal of Pileggi et al.^13^, adapted from the maternal near miss of the WHO, the following maternity and total indicators were calculated: a) rates of near miss (number of newborns meeting any of the validated criteria, but who have survived), early neonatal mortality, and severe outcomes (sum of cases of near miss and deaths) per 1,000 LB, to evaluate the burden of severe morbidity and mortality in maternity hospital at birth, and (b) percentage of infant mortality by components among cases of newborns with life-threatening conditions, which expresses lethality, to evaluate the need for investment in better perinatal care. We calculated neonatal and infant mortality rates and the ratio between near miss rates and mortality rates. We extended the calculation of all the indicators to 0–27 and 0–364 days, according to the definitions of near miss that, in the previous analysis, proved to be valid and adequate. The data were analyzed using the statistical package SPSS^®^, version 17, and Excel spreadsheet.

This study has been approved by the Research Ethics Committee of the Instituto de Estudos em Saúde Coletiva of the Universidade Federal do Rio de Janeiro (Process 15/2010) and the Faculdade de Saúde Pública of the Universidade de São Paulo (Process 2188/11), the Department of Health and Civil Defense of the Municipality of Rio de Janeiro (Process 87/2011), and the participating hospitals.

## RESULTS

Information on birth weight was present in all research records in the population eligible for analysis, 7,362 LB (Table 2). Gestational age was the most frequently incomplete information, followed by the Apgar score, although the magnitudes were less than 1%. As expected, pragmatic criteria with higher cutoff points increased the frequency of live births with life-threatening conditions. More children were born in this situation in the maternity hospital of Niterói, regardless of which of the four definitions of near miss was used to identify the presence of at least one pragmatic criterion. The lowest percentages of newborns with life-threatening conditions corresponded to the maternity hospitals of São Paulo (Table 2).

**Table 2:** Frequency of pragmatic criteria of life-threatening conditions and the presence of at least one of the criteria according to the definitions studieda. Municipalities of São Paulo, Rio de Janeiro, and Niterói, 2011.

Life threatening situation	Municipality of the public maternity hospitals							

	São Paulo		Rio de Janeiro		Niterói		Total	
			
	n = 5,579		n = 1,224		n = 559		n = 7,362	
Pragmatic criteria	n	%	n	%	n	%	n	%

Birth weight (BW-grams)								
< 1,500 g	56	1.0	17	1.4	17	3.0	90	1.2
< 1,750 g	98	1.8	28	2.3	29	5.2	155	2.1
Ignored	0	-	0	-	0	-	0	-
Gestational age (GA-weeks)								
< 30 w	45	0.8	11	0.9	11	2.0	67	0.9
< 31 w	56	1.0	14	1.1	15	2.7	85	1.2
< 32 w	70	1.3	20	1.6	18	3.2	108	1.5
< 33 w	97	1.7	28	2.3	26	4.7	151	2.1
Ignored	27	0.5	0	-	0	-	27	0.4
5’ Apgar score								
< 5	11	0.2	5	0.4	8	1.4	24	0.3
< 7	44	0.8	19	1.6	15	2.7	78	1.0
Ignored	18	0.3	2	0.2	2	0.4	22	0.3
At least one of the pragmatic criteria								
BW< 1,500 g or GA < 33 w or 5’ Apgar score < 7b	93	1.7	34	2.8	23	4.1	150	2.0
BW < 1,750 g or GA < 33 w or 5’ Apgar score < 7c	152	2.7	50	4.1	39	7.0	241	3.3
BW < 1,500 g or GA < 31 w or 5’ Apgar score < 5d	73	1.3	24	2.0	20	3.6	117	1.6
BW < 1,500 g or GA < 32 w or 5’ Apgar score < 7e	110	2.0	38	3.1	26	4.7	174	2.4
Ignored	43	0.8	2	0.2	2	0.4	47	0.6

^a^ The definitions of near miss will be named in this comparative study by the surname of the first author.

^b^ Pileggi et al.^13^

^c^ Pileggi-Castro et al.^14^

^d^ Souza et al.^20^

^e^ Silva et al.^16^

Then, we evaluated 7,315 LB that presented the three data: birth weight, gestational age, and fifth-minute Apgar score.

The frequency ranking of the isolated pragmatic criteria and their possible combinations to characterize the life-threatening situation showed variation, according to the definitions of near miss (Figure). In all definitions, except in Souza et al.^20^, the sum of the isolated criteria was greater than 50%. The combination of the criteria based on birth weight and gestational age most frequently classified newborns with life-threatening conditions, except for the definition of Pileggi et al.^13^, according to which the isolated criterion based on the Apgar score was predominant. No newborns presented the combination of Apgar score with birth weight and the combination of GA with Apgar score, only the near miss definition of Silva et al.^16^ (0.6%). The presence of the three pragmatic criteria occurred in smaller numbers, regardless of the definition of near miss.

**Figure:**
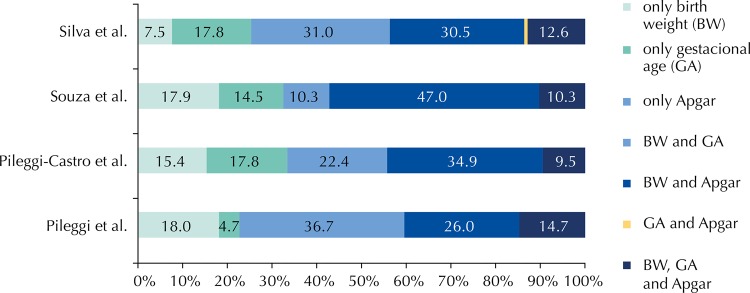
Frequency of pragmatic criteria and their combinations* according to definition of neonatal near miss. Municipalities of São Paulo, Rio de Janeiro, and Niterói, 2011.

* There was no case of near miss for the combinations of the pragmatic criteria of BW with GA and the combination of GA with Apgar escore, only to the near miss definition of Silva et al.^16^ (0.6%).

Pileggi et al.^13^: birth weight (BW < 1,500 g or gestational age (GA) < 30 weeks or 5’ Apgar score < 7.

Pileggi-Castrol et al.^14^: BW < 1,750 g or GA < 33 weeks or 5’ Apgar score < 7.

Souza et al.^20^: BW < 1,500 g or GA < 31 weeks or 5’ Apgar score < 5.

Silva et al.^16^: BW < 1,500 g or GA < 32 weeks or 5’ Apgar score < 7.

Table 3 shows the frequencies of newborns with life-threatening conditions, cases of near miss, and infant mortality by components and validation indicators according to the definition of near miss.

**Table 3:** Frequencies of newborn at risk, near miss cases by infant components, sensitivity, specificity, and confidence intervals (95%CI) according to definition of near miss. Municipalities of São Paulo, Rio de Janeiro, and Niterói, 2011.

Variable	Pragmatic criteria			

	Pileggi et al.^a^	Pileggi-Castro et al.^b^	Souza et al.^c^	Silva et al.^d^
Live births with life threatening condition	150	241	117	174

Early neonatal 0–6 days				
Cases of near miss	125	215	94	149
Sensitivity (%, 95%CI)	74.3 (57.8–86.0)	76.5 (59.8–87.8)	67.6 (50.8–81.0)	73.5 (56.7–85.6)
Specificity (%, 95%CI)	98.3 (98.0–98.6)	97.0 (96.6–97.4)	98.7 (98.4–99.0)	98.0 (97.6–98.3)
Neonatal 0–27 days				
Cases of near miss	116	205	85	140
Sensitivity (%, 95%CI)	72.3 (58.1–83.2)	76.6 (62.6–86.6)	68.1 (53.8–79.7)	72.3 (58.1–83.2)
Specificity (%, 95%CI)	98.4 (98.1–98.7)	97.2 (96.8–97.56)	98.8 (98.6–99.1)	98.1 (97.7–98.4)
Infant < 1 year				
Cases of near miss	110	198	80	134
Sensitivity (%, 95%CI)	58.8 (47.0–69.8)	63.2 (51.3–73.7)	54.4 (42.7–65.7)	58.8 (47.0–69.8)
Specificity (%, 95%CI)	98.5 (98.2–98.7)	97.3 (96.9–97.6)	98.9 (98.6–99.1)	98.2 (97.8–98.4)

^a^ Pileggi et al.^13^ (birth weight (BW) < 1,500 g or gestational age (GA) < 30 w or 5’ Apgar score < 7).

^b^ Pileggi-Castro et al.^14^ (BW < 1,750 g or GA < 33 w or 5’ Apgar score < 7).

^c^ Souza et al.^20^ (BW< 1,500 g or GA < 31 w or 5’ Apgar score < 5).

^d^ Silva et al.^16^ (BW < 1,500 g or GA < 32 w or 5’ Apgar score < 7).

When presenting at least one of the pragmatic criteria of the definition of near miss, 150 (2.1%), 241 (3.3%), 117 (1.6%), and 174 (2.4%) LB were classified as newborns with life threatening conditions, according to Pileggi et al.^13^, Pileggi-Castro et al.^14^, Souza et al.^20^, and Silva et al.^16^, respectively (Table 3).

There were 34 early neonatal deaths, 13 late neonatal deaths, and 21 post-neonatal deaths. Regardless of the infant age component, the sensitivity of the definition of near miss of Pileggi-Castro et al.^14^ was higher and, consequently, resulted in a higher number of false positives (LB at risk and survivors) (Table 3). As the definition of near miss of Souza et al.^20^ presented lower sensitivity, it classified a lower number of live births as cases of near miss. The definitions of near miss of Pileggi et al.^13^ and Silva et al.^16^ presented close sensitivity values (Table 3). For the four definitions, sensitivity remained the same or increased when the survival period was increased to 27 full days and decreased when the period was increased to up to under than one year of age. The highest sensitivity was the definition of Pileggi-Castro et al.^14^ in the neonatal period (76.6%), and the lowest sensitivity was the definition of Souza et al.^20^ among those under one year of age (54.4%). The specificity values were high (≥ 97%) and above the sensitivity values (approximately between 54% and 77%). The overlap of confidence intervals of sensitivity and specificity suggests that there is no statistically significant difference between the four definitions.

The indicators of birth and death and perinatal care evaluation are presented in Table 4. Maternity A (São Paulo) presented the best indicators: fewer births with life threatening conditions and lower rates of infant mortality, near miss, and severe outcomes, regardless of the definition of near miss. At the other extreme, maternity F (Niterói) presented the worst situation according to the indicators analyzed. We highlight that, in this maternity, more than half of the newborns with life threatening conditions according to the definition of near miss of Pileggi et al.^13^ died before reaching one year of age (Table 4).

**Table 4:** Live births and mortality rates by infant component and percentage of live births with life threatening condition, rates of neonatal near miss, severe outcome rates (1,000 live births), mortality rate among cases of near miss (%) according to definition of near miss and infant component. Municipalities of São Paulo, Rio de Janeiro, and Niterói, 2011.

Near miss	Indicator	Municipality/Maternity hospital						Total

		São Paulo				RJ	Niterói	
		
		A	B	C	D	E	F	
Total	Live births	1,621	1,592	749	1,574	1,222	557	7,315
	Mortality rate 0–6 days	1.9	6.9	2.7	4.4	2.5	14.4	4.1
	Mortality rate 0–27 days	2.5	8.8	2.7	5.7	3.3	25.1	6.4
	Mortality rate < 1 year	4.3	11.9	5.3	10.2	5.7	26.9	9.3
Pileggi et al.^a^	Live births with life-threatening condition (%)	0.4	2,6	1.6	2.1	2.8	4.1	2.1
0–6 days	Cases of near miss	6	35	10	26	31	17	125
	Near miss rate	3.7	22.0	13.4	16.5	25.4	30.5	17.1
	Severe outcome rate	5.6	28.9	16.0	21.0	27.8	44.9	21.7
	Death among newborns with life-threatening condition (%)	14.3	14.6	16.7	21.2	8.8	26.1	16.7
0–27 days	Cases of near miss	6	33	10	25	30	12	116
	Near miss rate	3.7	20.7	13.4	15.9	24.5	21.5	15.9
	Severe outcome rate	6.2	29.5	16.0	21.6	27.8	46.7	22.3
	Death among newborns with life-threatening condition (%)	14.3	19.5	16.7	24.2	11.8	47.8	22.7
< 1 year	Cases of near miss	6	30	9	25	29	11	110
	Near miss rate	3.7	18.8	12.0	15.9	23.7	19.7	15.0
	Severe outcome rate	8.0	30.8	17.4	26.0	29.5	46.7	24.3
	Death among newborns with life-threatening condition (%)	14.3	26.8	25.0	24.2	14.7	52.2	26.7
Silva et al.b	Live births with life-threatening condition (%)	0.7	2.9	1.9	2.5	3.1	4.7	2.4
0–6 days	Cases of near miss	10	40	12	32	35	20	149
	Near miss rate	6.2	25.1	16.0	20.3	28.6	35.9	20.4
	Severe outcome rate	8.0	32.0	18.7	24.8	31.1	50.3	25.0
	Death among newborns with life-threatening condition (%)	9.1	13.0	14.3	17.9	7.9	23.1	14.4
0–27 days	Cases of near miss	10	38	12	31	34	15	140
	Near miss rate	6.2	23.9	16.0	19.7	27.8	26.9	19.1
	Severe outcome rate	8.6	32.7	18.7	25.4	31.1	52.1	25.4
	Death among newborns with life-threatening condition (%)	9.1	17.4	14.3	20.5	10.5	42.3	19.5
< 1 year	Cases of near miss	10	35	11	31	33	14	134
	Near miss rate	6.2	22.0	14.7	19.7	27.0	25.1	18.3
	Severe outcome rate	10.5	33.9	20.0	29.2	32.7	52.1	27.6
	Death among newborns with life-threatening condition (%)	9.1	23.9	21.4	20.5	13.2	46.2	23.0

^a^ Pileggi et al.^13^: birth weight (BW) < 1,500 g or gestational age (GA) < 30 weeks or 5’ Apgar score < 7.

^b^ Silva et al.^16^: BW < 1,500 g or GA < 32 weeks or 5’ Apgar score < 7.

Using the definition of Silva et al.^16^, for every three survivors in life threatening situations, one neonatal death occurs in maternity A (São Paulo), approximately. This ratio increases to 3.6, 4.6, and 6.0 in maternities B, D, and C (São Paulo), respectively. In the state of Rio de Janeiro, the two municipalities represented in the study presented the extreme situations: in maternity E (Rio de Janeiro), the number of survivors with life threatening conditions was 11.7 times higher, while in maternity F (Niterói), it was only 2.5 times greater than the number of neonatal deaths.

## DISCUSSION

The validation of the pragmatic criteria of the four definitions of near miss in three different components of infant mortality, with data from this study, showed similar values of sensitivity and specificity. Based only on these results, we can infer that there is no statistically significant difference between the four definitions evaluated.

The cutoff points of the pragmatic criteria varied according to the definitions of near miss analyzed, mainly gestational age. Based on the strength of association with neonatal death, all were valid in the studies of neonatal near miss, including this study. However, we consider that the cutoff point at 32 weeks (including the categories of extremely preterm: ≤ 28 weeks and very preterm: > 28 and < 32 weeks^8^) is the most appropriate because it is based on different etiologies and interventions throughout gestation^18^ and because it is more frequently used than the other ones in epidemiological studies^4^ ^,^ ^7^ ^,^ ^8^ ^,^ ^11^ (< 30, < 31, and < 33). In a recent study using a systematic analysis of national databases and scientific articles, the global preterm estimate with less than 32 weeks was 15.6% for 1990–2010^4^. Children born at 32 weeks have little chance of survival in developing countries, but in developed countries, preterm survival at 32 weeks or more is similar to that of full-term children^3^.

Regarding birth weight, only the definition of Pileggi-Castro et al.^14^ had a cutoff point of 1,750 g, which resulted in greater sensitivity of the criterion, but it also a higher number of false positives. The cutoff point of 1,500 g and the corresponding classification, very low birth weight, have been used more frequently in epidemiological studies and perinatal risk classification^8^ ^,^ ^17^.

The Apgar score proposed 60 years ago, despite the possibility of incorporating the efficacy of immediate resuscitation measures between the first and fifth minutes when measured at the fifth minute, remains a strong predictor of infant mortality regardless of the presence of confounding and other risk factors^6^. The cutoff point of five is not used frequently in epidemiological studies, since the dichotomously analyzed Apgar score would include some degree of asphyxia in the lowest risk category, leading to underestimation. The most used intervals are: between seven and ten, considered normal; between zero and three, considered low; and between four and six, considered intermediate. The cutoff point of five for the fifth-minute Apgar score was less sensitive and more specific. In the study by Pileggi-Castro et al.^14^, when comparing the pragmatic criteria of the definitions of early neonatal near miss, the criterion of Souza et al.^20^, which is the only one with a cutoff point of five for the fifth-minute Apgar score, was less sensitive and more specific, similar to our results. Cutoff point seven was more adequate.

Periodic studies could apply a more complete definition, based on the literature on the subject and considering the availability of more technology in the care. When qualified information can be obtained on criteria other than the pragmatic ones, we recommend the incorporation of clinical, laboratory, and management criteria. The incorporation of congenital anomaly in the definition of near miss should be restricted to predictive anomalies of neonatal infant mortality, such as severe malformations of the central nervous system and congenital heart diseases, since most newborns with congenital anomalies will survive^2^, indicating the low specificity of this criterion. In addition, some limitations in relation to the complexity of the diagnosis and the time of registration may hinder the acquisition of information in maternity hospitals. Information on congenital anomalies in the Brazilian Live Birth Information System (SINASC) is restricted to apparent anomalies, verified by the professional responsible for the delivery^c^, often being of low risk for neonatal death, in addition to being underestimated^10^.

Regarding the survival period of the newborn of the four definitions of near miss analyzed, only the definition of Silva et al.^16^ reported the neonatal period of 0–27 days. The results of this study showed, in general, an increase in the sensitivity of the definition of near miss when comparing the periods of 0–6 and 0–27 complete days and a decrease in relation to the period of 0–364 days. In situations in which the monitoring of infant death after hospital discharge is not possible, we suggest the restriction to the early neonatal period or, otherwise, the adoption of the period of 0–27 days.

The indicators based on the pragmatic criteria of the definition of early neonatal near miss adequately reflected the care demand and intra-hospital care in hospitalization for deliveries regardless of the definition of neonatal near miss. We recommend their evaluation together, not in isolation.

In maternity F (Niterói), the burden of severe newborns (rate of severe outcomes) and the proportion of deaths among newborns with life-threatening conditions for the period of 0–6 full days were high, 50.3‰ LB and 23.1%, respectively (definition by Silva et al.^16^). Considering the high-risk care demand and the care demand from outside the municipality of the maternity hospital, this result seems to us to be consistent, reflecting issues related to the quality of intra-hospital care, which should be taken into account when defining priorities in the mother-child care^13^. Contrasting with maternity F, as expected, maternity A (São Paulo) presented the lowest burden of severe outcomes. We highlight that in maternity E (Rio de Janeiro), the burden of severe outcomes was relatively high (31.1‰), approximately four times the value of maternity A, but mortality among severe newborns (7.9%) was the lowest among all maternity hospitals evaluated.

The main advantages of the pragmatic criteria for the definition of near miss are the availability of information on birth weight, gestational age, and Apgar scores in the maternity hospitals and the SINASC and the ease to use the classification for clinical and epidemiological purposes. The completeness of the information from the pragmatic criteria in this study was excellent^15^, slightly higher than 99%. In addition to completeness, the information is available and easily obtainable. Accuracy and concordance of pregnancy outcomes (including birth weight, gestational age, and Apgar score of dichotomized medical record data) are generally considered as good and unbiased^1^. Brazilian studies have shown that birth weight^21^ and fifth-minute Apgar score showed good to excellent reliability^5^ ^,^ ^21^. The accuracy of gestational age, however, can be impaired by the measurement method itself^12^ ^,^ ^d^. Although the accuracy and reliability of these three variables were not analyzed in relation to different sources of information in this study, we believe that birth weight and fifth-minute Apgar score reproduced the pattern of improved validity and reproducibility, when compared to gestational age.

As in the study of Pileggi et al.^13^, in the maternity hospitals of São Paulo, the information on the gestational age analyzed was obtained from hospital records, which followed the protocols of the physicians who cared for the parturient, without mentioning the calculation or estimation method used. In the two maternity hospitals of the state of Rio de Janeiro, the researchers followed the algorithm of the hospitals, which prioritized DLP, when compatible with the US performed before 20 weeks, considered the most valid method^12^. In this sense, the measurement of gestational age was not standardized.

In addition, health indicators based on neonatal near miss allow us to monitor the quality of care provided and the care demand of a contingent of risky births, as well as identify children who will demand greater care from the health services and their family members.

One limitation of this study was the non-randomness of maternity hospitals and parturients, although the method used is similar to that used in the research of WHO^19^. In addition, the difficulties in relation to the greater accuracy and comparability of gestational age are also reported in several national and international studies^8^ ^,^ ^12^ ^,^ ^d^.

As sensitivity and specificity did not show significant differences, we recommend the use of the definition of Silva et al.^16^ because of the adequacy of the cutoff points with values currently used in perinatal studies. In addition, we emphasize that this definition of neonatal near miss was based on the analysis of exclusively Brazilian data and, therefore, more adequate to our reality.

We also recommend that, since individualized information can be obtained in the SINASC and SIM, for example, from the probabilistic relationship of the respective databases, or the hospital itself, indicators based on near miss should be monitored by a health establishment, contributing with the evaluation of the maternal and infant care.

## Footnotes

a Ministério da Saúde (BR), Secretaria de Vigilância em Saúde, Departamento de DST, Aids e Hepatites Virais. Painel de monitoramento da mortalidade infantil. Brasília (DF); 2017 [cited 2017 Jun 16]. Available from: http://svs.aids.gov.br/dashboard/mortalidade/infantil.show.mtw

b World Health Organization. Human Reproduction Programme: global survey on maternal and perinatal health. Geneva; c2017 [cited 2017 Jun 16]. Available from: http://www.who.int/reproductivehealth/topics/maternal_perinatal/globalsurvey/en/

c Ministério da Saúde (BR), Secretaria de Vigilância em Saúde, Departamento de Análise da Situação de Saúde. Manual de instruções para o preenchimento da Declaração de Nascido Vivo. Brasília (DF); 2011 [cited 2017 Jun 16]. (Série A, Normas e Manuais Técnicos). Available from: http://www.saude.ms.gov.br/wp-content/uploads/sites/88/2015/11/inst_dn.pdf

d National Institute for Health and Care Excellence. Antenatal care: routine care for the healthy pregnant woman. London: RCOG Press; 2008 [cited 2015 Feb 9]. (NICE Clinical Guideline, 62). Available from: http://www.nice.org.uk/nicemedia/pdf/CG062NICEguideline.pdf
